# Supplementary material for: Pyruvate Dehydrogenase Kinase 1 inhibition mediated oxidative phosphorylation enhancement in cartilage promotes osteoarthritis progression
Source: BMC Musculoskelet Disord. 2023 Jul 20;24:597. doi: 10.1186/s12891-023-06585-6 (PMC10357736; doi:10.1186/s12891-023-06585-6)
Supplement: Supplementary file 1 — Additional file 1. [file 12891_2023_6585_MOESM1_ESM.zip › KEGG.Gsea.1653622432464/KEGG_FRUCTOSE_AND_MANNOSE_METABOLISM.html]

Details for gene set KEGG\_FRUCTOSE\_AND\_MANNOSE\_METABOLISM[GSEA]

|  || Dataset | 55457\_collapsed\_to\_symbols.cls.cls #OA\_versus\_normal.cls.cls #OA\_versus\_normal\_repos |
| Phenotype | cls.cls#OA\_versus\_normal\_repos |
| Upregulated in class | OA |
| GeneSet | KEGG\_FRUCTOSE\_AND\_MANNOSE\_METABOLISM |
| Enrichment Score (ES) | 0.23376736 |
| Normalized Enrichment Score (NES) | 0.83896035 |
| Nominal p-value | 0.7297921 |
| FDR q-value | 0.922154 |
| FWER p-Value | 1.0 |
Table: GSEA Results Summary

  

Fig 1: Enrichment plot: KEGG\_FRUCTOSE\_AND\_MANNOSE\_METABOLISM      
 Profile of the Running ES Score & Positions of GeneSet Members on the Rank Ordered List

  

| SYMBOL | TITLE | RANK IN GENE LIST | RANK METRIC SCORE | RUNNING ES | CORE ENRICHMENT || 1 | MPI | mannose phosphate isomerase [Source:HGNC Symbol;Acc:HGNC:7216] | 255 | 0.781 | 0.0841 | Yes |
| 2 | FBP1 | fructose-bisphosphatase 1 [Source:HGNC Symbol;Acc:HGNC:3606] | 772 | 0.576 | 0.1207 | Yes |
| 3 | ALDOC | "aldolase, fructose-bisphosphate C [Source:HGNC Symbol;Acc:HGNC:418]" | 870 | 0.551 | 0.1866 | Yes |
| 4 | PFKM | "phosphofructokinase, muscle [Source:HGNC Symbol;Acc:HGNC:8877]" | 2260 | 0.331 | 0.1227 | Yes |
| 5 | KHK | ketohexokinase [Source:HGNC Symbol;Acc:HGNC:6315] | 2809 | 0.269 | 0.1160 | Yes |
| 6 | AKR1B1 | aldo-keto reductase family 1 member B [Source:HGNC Symbol;Acc:HGNC:381] | 3138 | 0.236 | 0.1220 | Yes |
| 7 | FPGT | fucose-1-phosphate guanylyltransferase [Source:HGNC Symbol;Acc:HGNC:3825] | 3259 | 0.225 | 0.1427 | Yes |
| 8 | PFKFB2 | "6-phosphofructo-2-kinase/fructose-2,6-biphosphatase 2 [Source:HGNC Symbol;Acc:HGNC:8873]" | 3547 | 0.198 | 0.1468 | Yes |
| 9 | FBP2 | fructose-bisphosphatase 2 [Source:HGNC Symbol;Acc:HGNC:3607] | 3763 | 0.180 | 0.1540 | Yes |
| 10 | HK3 | hexokinase 3 [Source:HGNC Symbol;Acc:HGNC:4925] | 3792 | 0.178 | 0.1755 | Yes |
| 11 | TPI1 | triosephosphate isomerase 1 [Source:HGNC Symbol;Acc:HGNC:12009] | 4059 | 0.150 | 0.1748 | Yes |
| 12 | GMDS | "GDP-mannose 4,6-dehydratase [Source:HGNC Symbol;Acc:HGNC:4369]" | 4151 | 0.142 | 0.1867 | Yes |
| 13 | SORD | sorbitol dehydrogenase [Source:HGNC Symbol;Acc:HGNC:11184] | 4231 | 0.134 | 0.1984 | Yes |
| 14 | PMM1 | phosphomannomutase 1 [Source:HGNC Symbol;Acc:HGNC:9114] | 4238 | 0.134 | 0.2157 | Yes |
| 15 | ALDOA | "aldolase, fructose-bisphosphate A [Source:HGNC Symbol;Acc:HGNC:414]" | 4289 | 0.129 | 0.2290 | Yes |
| 16 | MTMR7 | myotubularin related protein 7 [Source:HGNC Symbol;Acc:HGNC:7454] | 4432 | 0.119 | 0.2338 | Yes |
| 17 | PFKP | "phosphofructokinase, platelet [Source:HGNC Symbol;Acc:HGNC:8878]" | 5500 | 0.032 | 0.1552 | No |
| 18 | PFKFB4 | "6-phosphofructo-2-kinase/fructose-2,6-biphosphatase 4 [Source:HGNC Symbol;Acc:HGNC:8875]" | 5534 | 0.030 | 0.1566 | No |
| 19 | PFKL | "phosphofructokinase, liver type [Source:HGNC Symbol;Acc:HGNC:8876]" | 5632 | 0.021 | 0.1519 | No |
| 20 | MTMR2 | myotubularin related protein 2 [Source:HGNC Symbol;Acc:HGNC:7450] | 6189 | -0.019 | 0.1113 | No |
| 21 | HK1 | hexokinase 1 [Source:HGNC Symbol;Acc:HGNC:4922] | 6728 | -0.058 | 0.0772 | No |
| 22 | GMPPA | GDP-mannose pyrophosphorylase A [Source:HGNC Symbol;Acc:HGNC:22923] | 7039 | -0.083 | 0.0641 | No |
| 23 | GFUS | GDP-L-fucose synthase [Source:HGNC Symbol;Acc:HGNC:12390] | 7317 | -0.103 | 0.0563 | No |
| 24 | MTMR1 | myotubularin related protein 1 [Source:HGNC Symbol;Acc:HGNC:7449] | 8102 | -0.163 | 0.0172 | No |
| 25 | AKR1B10 | aldo-keto reductase family 1 member B10 [Source:HGNC Symbol;Acc:HGNC:382] | 8420 | -0.187 | 0.0174 | No |
| 26 | ALDOB | "aldolase, fructose-bisphosphate B [Source:HGNC Symbol;Acc:HGNC:417]" | 8421 | -0.187 | 0.0423 | No |
| 27 | PMM2 | phosphomannomutase 2 [Source:HGNC Symbol;Acc:HGNC:9115] | 9408 | -0.265 | 0.0009 | No |
| 28 | HK2 | hexokinase 2 [Source:HGNC Symbol;Acc:HGNC:4923] | 10030 | -0.325 | -0.0041 | No |
| 29 | MTMR6 | myotubularin related protein 6 [Source:HGNC Symbol;Acc:HGNC:7453] | 11085 | -0.442 | -0.0271 | No |
| 30 | PFKFB1 | "6-phosphofructo-2-kinase/fructose-2,6-biphosphatase 1 [Source:HGNC Symbol;Acc:HGNC:8872]" | 11840 | -0.557 | -0.0115 | No |
| 31 | PFKFB3 | "6-phosphofructo-2-kinase/fructose-2,6-biphosphatase 3 [Source:HGNC Symbol;Acc:HGNC:8874]" | 12388 | -0.704 | 0.0398 | No |
Table: GSEA details [plain text format]

  

Fig 2: KEGG\_FRUCTOSE\_AND\_MANNOSE\_METABOLISM      
 Blue-Pink O' Gram in the Space of the Analyzed GeneSet

  

Fig 3: KEGG\_FRUCTOSE\_AND\_MANNOSE\_METABOLISM: Random ES distribution      
 Gene set null distribution of ES for **KEGG\_FRUCTOSE\_AND\_MANNOSE\_METABOLISM**
